# An Agar-Free, Glass Bead-Based Method for the Culture of *Strongyloides stercoralis*: An Exploratory Diagnostic Sensitivity Study

**DOI:** 10.3390/diagnostics16050711

**Published:** 2026-02-27

**Authors:** Francesca Tamarozzi, Monica Degani, Salvatore Scarso, Sara Negrelli, Stefano Tais, Eleonora Rizzi, Alberta Carrara, Giulia La Marca, Davide Treggiari, Tamara Ursini, Dora Buonfrate

**Affiliations:** Department of Infectious Tropical Diseases and Microbiology, IRCCS Sacro Cuore Don Calabria Hospital, 37024 Negrar di Valpolicella, Verona, Italy; monica.degani@sacrocuore.it (M.D.); salvatore.scarso@sacrocuore.it (S.S.); sara.negrelli@sacrocuore.it (S.N.); stefano.tais@sacrocuore.it (S.T.); eleonora.rizzi@sacrocuore.it (E.R.); alberta.carrara@sacrocuore.it (A.C.); giulia.lamarca@sacrocuore.it (G.L.M.); davide.treggiari@sacrocuore.it (D.T.); tamara.ursini@sacrocuore.it (T.U.); dora.buonfrate@sacrocuore.it (D.B.)

**Keywords:** *Strongyloides stercoralis*, strongyloidosis, diagnosis, culture, PCR

## Abstract

**Background/Objectives**: The diagnosis of infection with *Strogyloides stercoralis*, recently targeted for control by the World Health Organization, (WHO), is challenging. Specific coproparasitological methods (agar plate culture [APC], Baermann sedimentation), recommended by the WHO for public health use, are labor-intensive and require bulky disposable materials as well as experienced microscopists. We explored the sensitivity of an alternative stool culture method using recyclable glass beads, followed by microscopy and PCR in comparison to routine APC and PCR performed on uncultured stool. **Methods**: We conducted a diagnostic sensitivity study on samples from patients with positive serology for strongyloidiasis who submitted stool specimens to our laboratory between January 2023 and December 2025 for parasitological confirmation. Samples were processed by routine APC and PCR on fresh stool, as well as experimental culture on bead-based plates (BPC), PCR on APC- and BPC-cultured stool, and PCR on stool incubated directly in the collection container. **Results**: Twenty-six of 110 samples (23.6%) tested positive in at least one technique. Within this subset, the most sensitive techniques were the APC and PCR after APC (both 84.62%); PCR on fresh stool was the least sensitive (42.31%) (*p* = 0.002). The sensitivity of BPC (65.38%) was lower than that of APC, although not statistically significantly. Comparable sensitivity was observed between microscopy and PCR after APC or BPC. PCR after incubation in the container showed a sensitivity of 57.69%. Agreement ranged from 50 to 84.6%. **Conclusions**: Alternative culture methods with more field-friendly implementation features could be interesting alternatives to standard methods. Further studies evaluating their performance and applicability in public health and clinical contexts are warranted.

## 1. Introduction

Strongyloidiasis is an infection caused by the soil-transmitted helminth *Strongyloides stercoralis* [1]. It is estimated that over 600 million people are infected worldwide, mostly in tropical and subtropical regions [2]. Infection is acquired through percutaneous penetration of infective third-stage larvae (L3) present in soil contaminated with infected human feces, and may theoretically persist across one’s lifetime due to the parasite’s peculiar auto-infective cycle [1]. Although most infections are asymptomatic or cause unspecific gastrointestinal, respiratory, and cutaneous signs and symptoms of variable severity, hyperinfection and dissemination may develop in immunocompromised individuals, and are frequently fatal [1]. The World Health Organization (WHO) has recently recommended that endemic countries map strongyloidiasis and assess the need for public health control interventions [3].

The diagnosis of infection with *S. stercoralis* is challenging [4,5]. No diagnostic-reference standard assay with both high sensitivity and high specificity is available, and determining infection status is complex, with infected individuals being variably classified as positive or negative by different assays (serology, coproparasitology, molecular assays) [6,7,8,9,10,11,12]. Standard coproparasitological assays (Kato-Katz, flotation, formol-ether concentration) are unreliable for *S. stercoralis* [4,13,14,15,16,17,18]. Specific direct methods for the retrieval and identification of parasite larvae in stool by microscopy (agar plate culture [APC], Baermann sedimentation) or by detection of parasite DNA are currently considered the best reference assays for the diagnosis of infection. These techniques are highly specific but only moderately sensitive (generally in the range of 40–80%) [4,18,19,20]. Among these techniques, APC generally demonstrates higher sensitivity (generally in the range of 60–98%) [4] due to the amplification of the parasite load obtained from the development of free-living adults followed by second-generation L3 in a multiple-day culture.

Coprological methods such as the Baermann technique or APC are the assays recommended by the World Health Organization (WHO) guidelines on preventive chemotherapy for public health control of strongyloidiasis to determine community-level infection prevalence and guide decision making [3]. However, these specific parasitological methods are labor-intensive and require bulky disposable materials [4,6,21]. Recently, the Target Product Profiles (TPP) for new diagnostics to inform strongyloidiasis control programs were developed under WHO guidance [22,23]. This document provides minimum and ideal technical (design, configuration, cost, procurement) and performance characteristics for diagnostics intended for public health use. Within this framework, stool culture methods using fully recyclable materials could facilitate the deployment of these techniques. Moreover, coupling culture with PCR could allow omission of the microscopy step, which requires experienced microscopists able to distinguish *S. stercoralis* larvae from those of other parasites (e.g., hookworm).

The primary aim of this pilot study was to explore the sensitivity of an alternative stool culture method using recyclable glass beads, followed by microscopy and PCR, for the diagnosis of *S. stercoralis* infection, in comparison with routine APC and PCR performed on uncultured stool.

## 2. Materials and Methods

### 2.1. Study Design and Patient Selection

This study was designed as a diagnostic sensitivity study. The study was embedded within the routine workflow for the diagnosis of strongyloidiasis at the Department of Infectious-Tropical Diseases and Microbiology (DITM) of IRCCS Sacro Cuore Don Calabria Hospital, WHO Collaborating Centre on Strongyloidiasis and other Neglected Tropical Diseases. Routine screening for strongyloidiasis is performed by serology using the *Strongyloides* IgG ELISA (Bordier Affinity Products, Crissier, Switzerland). All patients with positive serological tests are prompted to provide a fresh stool sample to perform APC and PCR. This study was performed on samples from all consecutive patients with positive serological results for strongyloidiasis who submitted fresh stool samples to the parasitology laboratory of DITM between January 2023 and December 2025 for parasitological confirmation whose samples were of an adequate quantity to perform all study laboratory procedures.

Ethical review and approval were waived for this study. All patients submitting biological samples for diagnostic purposes to the parasitology laboratory of DITM provide written and signed consent to the anonymized use of residual material for research, which was used for this study. No additional samples were requested for this study and the results of the experimental methods did not impact the clinical decision to treat the patients since all patients with positive serological results for strongyloidiaisis were treated with ivermectin regardless of the result of parasitological assays. The study was conducted according to the Declaration of Helsinki and its updates.

### 2.2. Laboratory Procedures

**Figure 1:**
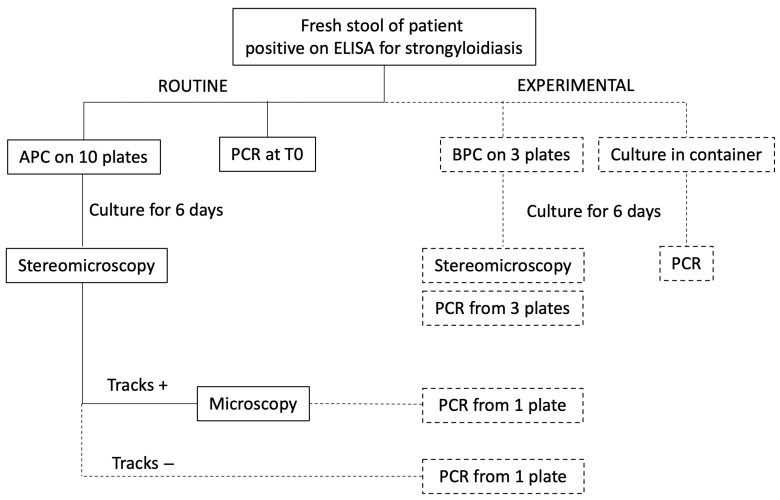
Study workflow. Routine diagnostic procedures are indicated with continuous lines and boxes. Experimental procedures are indicated with dashed lines and boxes. APC: agar plate culture. BPC: bead plate culture. PCR at T0: PCR on fresh stool. Tracks: bacterial colonies growing on the path followed by crawling larvae on agar.

The study workflow is schematized in Figure 1.

Routine APC was performed by plating 5 g of fresh, unpreserved and unrefrigerated stool mixed with vegetable charcoal in a 2:1 ratio on each agar plate for culturing *Strongyloides* (Biolife italiana Srl, Milan, Italy). A total of 10 plates were cultured for each sample. Plates were incubated at 26 °C for 6 days. Culture negativity or morphological identification of larvae [4] was defined by observation of tracks (bacterial colonies growing on the path formed by crawling larvae on agar) by stereomicroscopy. The surfaces of plates with visible tracks were then rinsed with 10% formalin, and the sediment examined by optical microscopy by an experienced microscopist. A sample was classified as positive if at least one agar plate was positive for the presence of *S. stercoralis* larvae.

Routine real-time (RT)-PCR was performed on fresh stool based on the protocol of Verweij et al. [19]. Centrifugations were carried out in a centrifuge with a 20 cm radius rotor. One gram of fresh stool was fixed in 4 mL of 70% ethanol. For pre-extraction of DNA, 0.5 mL of ethanol-fixed stool was added to tubes containing beads (MagnaLyzer Green Beads Roche, Basel, Switzerland) together with 100 µL of S.T.A.R. buffer (Roche, Basel, Switzerland) and 4 µL of internal Phocid alphaherpesvirus 1 (PhHV-1 [24]) control. This resulted in a final approximate threshold cycle (Ct) value of the internal control of 25–30. Samples were centrifuged in the MagnaLyzer (Roche) at 3000× rpm for 30 s, followed by a brief centrifugation at 10,000× rpm for 10 s. Samples were then incubated at 95 °C for 10 min in a thermal block, vortexed for 10 s, and centrifuged for 1 min at 10,000× rpm. Then, 150 µL of each sample was diluted in 450 µL of nuclease-free water using the Hamilton MagEx system (Hamilton Company, Reno, NV, USA). DNA extraction was performed using the MagMAX™ Viral/Pathogen Nucleic Acid Isolation Kit (Thermo Fisher Scientific, Waltham, MA, USA) as per the manufacturer’s instructions. Nucleic acids were eluted at room temperature in 100 µL of the kit’s elution buffer. PCR reactions were set up at a 25 μL volume, containing 2×SsoFast Mastermix (Bio-Rad, Hercules, CA, USA), 2.5 μg BSA (Sigma-Aldrich, St Louis, MO, USA), 80 nM of each PhHV-1 primer and 200 nM of each probe, 100 nM of each *S. stercoralis* primer and probe, and 5 μL of sample DNA. PCR was performed on a CFX Real-Time System (Bio-Rad) under the following cycling conditions: 95 °C for 3 min, 40 cycles of 95 °C for 15 s, 60 °C for 30 s, and 72 °C for 30 s. The Ct output was analyzed using BioRad CFX Maestro 1.1 software (version 4.1.2433.1219). A Ct ≤ 40 with amplification signals with the typical morphology of a RT-PCR amplification curve was considered a positive result. Runs with no amplification of internal controls were considered as not determined.

For the experimental culture on beads (bead-based plate culture—BPC), 3 mm diameter glass beads (BBTrade, Besana in Brianza, Italy) were placed in an empty 9.2 cm diameter Petri dish (Vizaplastik Diagnostici s.r.l., Vicenza, Italy), together with 4 mL tap water, to form a loose monolayer. Then, 5 g of the charcoal-mixed stool sample was plated onto each of three 6 cm diameter blotting paper disks (Sanitaria Corato, Verona, Italy). The disks were then placed on top of the beads and incubated at 26 °C for 6 days. Culture results were classified as positive based on the presence of larvae observed by stereomicroscopy. A sample was considered positive if at least one plate showed the presence of *S. stercoralis* larvae.

Finally, the remaining charcoal-mixed stool in the original collection container was incubated for 6 days at 26 °C with the lid loosely opened.

At the end of the 6-day incubation period, PCR was performed on cultured samples. For APC cultures, samples for PCR were obtained either from one plate showing visible tracks on stereomicroscopy or from one randomly selected plate if none of the 10 agar plates showed tracks. The plate surface was washed with 4 mL 70% ethanol; the wash was collected in a tube and added with 1 g of fecal material from the same plate. For the BPC cultured samples, approximately 30 mg of cultured feces were collected from each of the three plates, pooled (to obtain 1 g feces in total) and transferred into a tube containing 4 mL of 70% ethanol. Finally, an aliquot of 1 g charcoal-mixed stool from the original stool sample container incubated for 6 days was also collected and placed in a tube containing 4 mL of 70% ethanol. The ethanol-fixed stool samples were subsequently used to perform PCR, as described above.

### 2.3. Data Analysis

The study sample was a convenience sample, determined by the number of eligible stool samples available during the study period. Data were summarized as counts and percentages. The sensitivity of each method was calculated as the percentage of positive results on the total number of samples that tested positive in at least one fecal technique (i.e., any culture or PCR method), with corresponding 95% confidence intervals (CI). Sensitivities were compared using Cochrane’s Q test and McNemar’s test with Bonferroni correction for multiple comparisons. Agreement between methods was assessed on the entire sample set and on the subset of samples positive in at least one technique (culture or PCR), using Gwet’s AC1 coefficient and Cohen’s K coefficient. Agreement was classified as follows: poor (<0.2), fair (0.21–0.40), moderate (0.41–0.60), substantial (0.61–0.80), and very good (>0.80). All analyses were performed using Excel (Microsoft) with Real Statistics Resource Pack (https://real-statistics.com/).

## 3. Results

**Figure 2:**
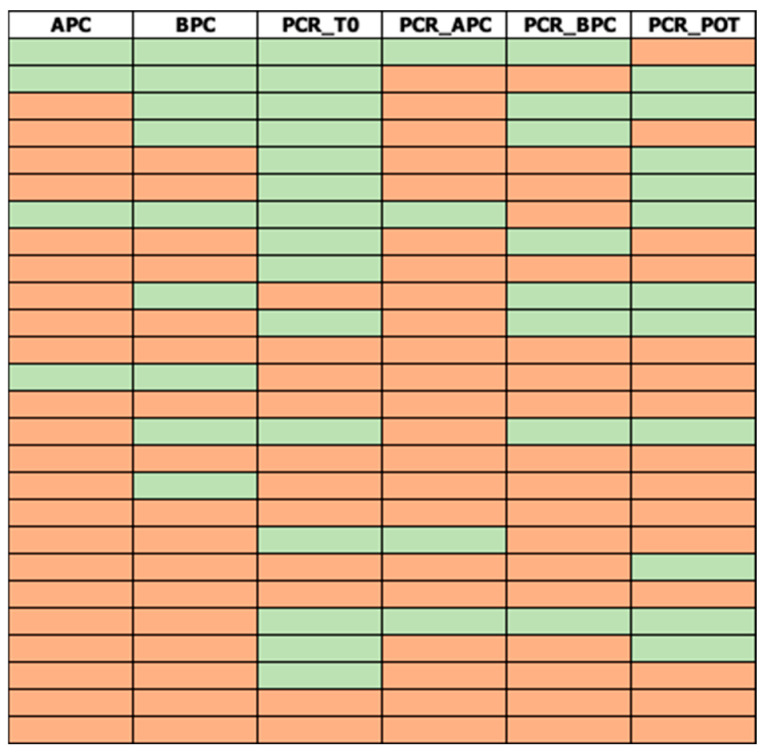
Heat map representing the positivity (red) or negativity (green) of each sample processed with each method, among samples positive in at least one technique (N = 26). Samples negative for all techniques (N = 84) are omitted from the graph. APC: agar plate culture. BPC: bead plate culture. PCR-T0: PCR on fresh stool. PCR_APC: PCR from APC. PCR_BPC: PCR from bead plate culture. PCR_POT: PCR from culture in the stool container.

In the study period, 110 patients with positive serology for strongyloidiasis submitted fresh stool samples for parasitological and molecular analyses. A summary of the results of each technique applied is presented in Table 1; the raw data are available as Supplementary File S1. Eighty-four samples (84/110; 76.4%) tested negative for all techniques, while 26/110 (23.6%) were positive for at least one technique (Figure 2). No PCR runs failed the amplification of internal controls.

**Figure 3:**
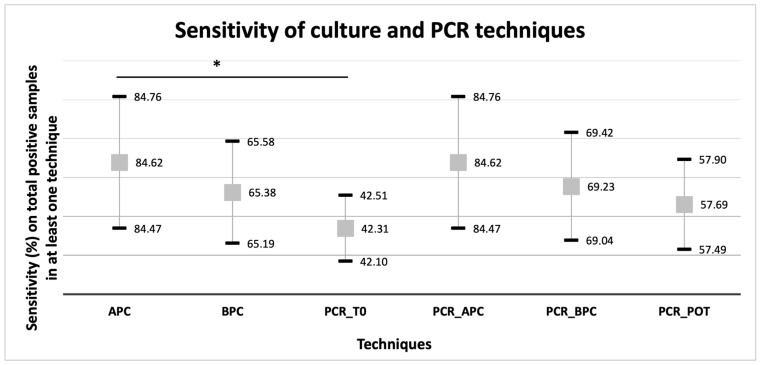
Sensitivity (%) of each technique on the total samples positive for *S. stercoralis* for at least one technique (N = 26). Gray boxes represent the percentage of positive results on the total number of samples positive in at least one technique. Error bars indicate 95% confidence intervals. * Statistically significant difference after Bonferroni correction (threshold for statistical significance: *p*-value = 0.005).

In the subset of samples positive in at least one culture or PCR method, the most sensitive techniques were the routine APC and PCR from APC (both 84.62%; 95%CI 84.47–84.76), whereas PCR on fresh stool was the least sensitive (42.31%; 95%CI 42.10–42.51) (Figure 3). A significant difference in the percentages of positivity was observed among the groups (Q = 19.599; *p*-value = 0.001). In pairwise comparisons with Bonferroni correction, statistical significance (*p*-value <0.005) was reached only by APC vs. PCR performed on fresh stool at diagnosis (X2 = 9.308, *p*-value = 0.002). Combining any two methods did not result in the detection of all 26 positive cases.

Calculations of agreement between tests are provided in Supplementary File S1. When concordance of methods was assessed across the whole cohort of 110 samples, percent agreement ranged between 88.2 and 96.4%. Concordance was very good for all pairwise comparisons using Gwet’s AC1 coefficient (range 0.841–0.947), whereas, as expected, results were more variable when using Cohen’s Kappa, but remained moderate to substantial in all comparisons (range 0.512–0.886). However, this good level of concordance was largely driven by the large proportion of samples (84/110; 76.4%) that tested negative across all methods. When the analysis was restricted to the subset of samples positive in at least one method, agreement was considerably lower. Percent agreement ranged from 50 to 84.6%; the highest concordance, rated as moderate to substantial, was obtained for APC and BPC (agreement 80.0% [95%CI 64.9–96.7]; Cohen’s Kappa 0.51 [95%CI 0.16–0.86]; Gwet’s AC1 0.69 [95%CI 0.41–0.98]), and for APC and PCR on APC-cultured stool (agreement 84.6% [95%CI 70.0–99.2]; Cohen’s Kappa 0.41 [95%CI 0.00–0.91]; Gwet’s AC1 0.79 [95%CI 0.57–1]).

An exploratory analysis was conducted to compare the sensitivity of APC and BPC under the hypothetical scenario in which only a single plate was cultured per sample. Data on the number of positive plates on APC culture and BPC culture were available for only 15/22 (68.2%) and 10/17 (58.8%) positive cultures, respectively. The majority (10/15; 66.7%) of APC-positive samples had all 10 plates developing larvae, with the minimum number of positive plates being two. For BPC, the majority (8/10; 80%) of positive samples had all three plates develop larvae, while two samples had only one positive plate. When exploratorily applying a probabilistic approach based on the hypergeometric distribution to this limited subset of data, the sensitivity of a single-plate APC and BPC followed by microscopy was 87.3% and 86.7%, respectively.

## 4. Discussion

We conducted a pilot study to explore the comparative sensitivity of alternative direct coprological methods for the diagnosis of *S. stercoralis* infection, which could facilitate their application by simplifying culture material requirements and potentially eliminating the need for microscopy. Indeed, currently recommended techniques for the diagnosis of *S. stercoralis* infection (APC, Baermann) require the use of bulky, non-recyclable material, which pose challenges in procurement, cost, and disposal. Furthermore, these methods depend on microscopy, which is time-consuming and requires experienced microscopists capable of distinguishing *S. stercoralis* from other nematodes that might be present in human stool (e.g., hookworm).

With the limits derived from the study design (see below), APC performed on a large stool sample volume was demonstrated to be the most sensitive technique, consistent with the literature [4]. When evaluating the bead-based culture method (BPC) developed at DITM, which is agar-free and applicable with reusable material, the sensitivity of this method for the total of samples positive for at least one fecal technique was 65.38% ± 4.9%. This was lower than that of APC, although the difference was not statistically significant after Bonferroni correction (*p*-value = 0.03; Bonferroni-adjusted threshold *p*-value = 0.005). This sensitivity exceeded that reported for other filter paper-based techniques described in the literature for *S. stercoralis* (reported in the range 19–57%) [4,15,25,26,27,28]. However, direct comparison is challenging due to protocol heterogeneity. Most importantly, this sensitivity is higher than the minimum required sensitivity of 50% identified by the WHO for tests to be used in the public health context [22,23]. Moreover, importantly, this level of sensitivity was achieved using less than 30% of the stool volume used for APC (3 plates with BPC vs. 10 plates for APC).

Stool culture, which allows the development of free-living adult *S. stercoralis* followed by a new generation of larvae, amplifies parasite load in a sample, as shown also by our results: the number of positive samples approximately doubled when PCR was performed on cultured compared with uncultured stool. For both APC and BPC, comparable sensitivity was found for microscopy and PCR performed on cultured samples. These results suggest that PCR on cultured feces could obviate the need for microscopy when classical parasitology is unavailable or impractical and molecular diagnosis is instead an option, achieving similarly high sensitivity. Although PCR on samples obtained from any culture technique achieved higher sensitivity than PCR on fresh stool, the gain in sensitivity compared to PCR on fresh stool was only marginal when stool was just mixed with charcoal and incubated in the sample collection container rather than on agar or bead plates. Although this is unfortunate, as this approach would have minimized material use, the sensitivity achieved (57.69% ± 0.20%) was still higher than the minimum required sensitivity of 50% identified by the WHO TPP for *S. stercoralis* diagnostics for a test to be used in the public health context [22,23]. Careful modeling of survey costs incorporating molecular analysis instead of microscopy on stool cultured with different methods is required to evaluate the cost-efficiency of these alternative strategies [22].

This study has several limitations. First, the sample size was small and, owing to the relatively low frequency and unpredictable influx of patients with strongyloidiasis, was determined by logistical and time constraints rather than formal statistical calculations. In addition, only samples from patients with positive serological results for strogyloidiasis were included, due to the routine diagnostic workflow in our structure. Consequently, sensitivity estimates obtained in this study may not reflect the values that would be obtained if these assays were used as the sole diagnostic method, and should therefore be considered exploratory and interpreted primarily in comparative terms. It was not possible to apply statistical approaches such as latent class analysis to estimate sensitivity in the absence of a gold standard while accounting for false-positive and false-negative serology results [29]. Therefore, further studies applying, in parallel, multiple diagnostic techniques combined with appropriate statistical analyses are required to confirm whether sensitivities meet WHO thresholds for public health deployment. Additionally, the study was most likely underpowered to detect statistically significant differences between groups.

Second, the two culture methods and the material used for PCR on cultured stool were not directly comparable. APC was performed on 10 plates with positivity on microscopy primarily guided by the visualization of tracks, whereas BPC was performed on three plates and positivity was not guided by visualization of tracks (which were not visible with this system). This difference in set-up was due to the need to embed the study within the routine diagnostic workflow of the DITM parasitology laboratory. While 10 APC plates were always cultured, as this constitutes the standard routine diagnostic at DITM, it was not always possible to obtain an adequate stool sample volume to also perform the other methods. Based on experience, it was judged that in most cases, only three extra plates could be cultured reliably from the available stool samples. In addition, material to perform PCR on APC was sampled from a single plate, while for BPC, pooled stool from the three plates was used. On one hand, it can be speculated that the lower sensitivity observed with BPC was at least partly due to the smaller stool sample volume processed with this method compared with APC. On the other hand, the sensitivity of APC might have been lower than that which would have been obtained if all plates, with and without visible tracks, were washed and processed by microscopy.

The WHO guidelines on preventive chemotherapy for public health control of strongyloidiasis indicate that Baermann sedimentation or APC from a single stool specimen should be used for diagnosis in public health settings [3]. Although the guidelines do not specify the number of agar culture plates to be seeded from this single stool specimen, seeding of a single plate is the most likely implementable approach [21]. Therefore, we conducted a theoretical exploration of the performance of a single-plate APC and BPC approach followed by microscopy. This was an exploratory analysis, and the results should be interpreted with caution. Our findings suggest that the sensitivities of single-plate APC and BPC are comparable, but direct head-to-head comparison is required for confirmation. Furthermore, further studies should compare head to head single-plate APC and BPC cultures followed by PCR, and potentially Baermann sedimentation, to fully evaluate the methods’ performances.

## 5. Conclusions

This pilot study explored the comparative sensitivity of alternative direct coprological methods for the diagnosis of *S. stercoralis* infection. These methods may facilitate their implementation in different settings, ease material procurement and disposal, or allow replacement of microscopy with PCR where desirable. We found that alternative culture methods (recyclable agar-free bead-based culture; simple stool incubation with charcoal) could be interesting alternatives to standard methods, warranting further evaluation of their performance and feasibility alongside routine methods, in both public health and clinical diagnostic contexts. To this end, cross-sectional and cohort studies applying multiple diagnostic techniques in parallel, together with statistical methods suitable for estimating accuracy in the absence of a gold standard, are required.

## Figures and Tables

**Table 1:** Summary of results from the applied techniques. APC: agar plate culture. BPC: bead plate culture. PCR-T0: PCR on fresh stool. PCR_APC: PCR from APC. PCR_BPC: PCR from bead plate culture. PCR_POT: PCR from culture in the stool container.

	Overall	APC	BPC	PCR_T0	PCR_APC	PCR_BPC	PCR_POT
**N positive** **(%)**	26/110 (23.6%)	22/110 (20%)	17/110 (15%)	11/110 (10%)	22/110 (20%)	18/110 (16%)	15/110 (13%)
**N negative ** **(%)**	84/110 (76.4%)	88/110 (80%)	93/110 (85%)	99/110 (90%)	88/110 (80%)	92/110 (84%)	95/110 (87%)

## Data Availability

The original contributions presented in this study are included in the article/Supplementary Material. Further inquiries can be directed to the corresponding author.

## Supplementary Materials

The following supporting information can be downloaded at https://www.mdpi.com/article/10.3390/diagnostics16050711/s1. File S1: original data and tests agreement analysis.

## Abbreviations

The following abbreviations are used in this manuscript:

APC	Agar Plate Culture
BPC	Bead-Based Plate culture
CI	Confidence Interval
DITM	Department of Infectious Tropical Diseases and Microbiology of IRCCS Sacro Cuore Don Calabria Hospital, 37024 Negrar di Valpolicella, Verona, Italy
TPP	Target Product Profile
WHO	World Health Organization
